# Junctional Modulation of Round Window Membrane Enhances Dexamethasone Uptake into the Inner Ear and Recovery after NIHL

**DOI:** 10.3390/ijms221810061

**Published:** 2021-09-17

**Authors:** Seong-Hun Jeong, Yoonjoong Kim, Ah-Ra Lyu, Sun-Ae Shin, Tae Hwan Kim, Yang Hoon Huh, A Reum Je, Akanksha Gajibhiye, Yang Yu, Yongde Jin, Min Jung Park, Yong-Ho Park

**Affiliations:** 1 Department of Medical Science, Chungnam National University, Daejeon 35015, Korea; hunpass2@gmail.com (S.-H.J.); ahmilove@naver.com (A.-R.L.); akanksha0323@gmail.com (A.G.); 2 Department of Otolaryngology—Head and Neck Surgery, Chungbuk National University Hospital, Cheongju 28644, Korea; mdyjkim@gmail.com; 3 Department of Otolaryngology—Head and Neck Surgery, Chungnam National University, Daejeon 35015, Korea; ttd0707@naver.com (S.-A.S.); ent-yuyang0924@naver.com (Y.Y.); 4 Brain Research Institute, College of Medicine, Chungnam National University, Daejeon 35015, Korea; 5 Biomedical Research Institute, Chungnam National University Hospital, Daejeon 35015, Korea; czkth@naver.com; 6 Electron Microscopy Research Center, Korea Basic Science Institute, Cheongju 28116, Korea; hyh1127@kbsi.re.kr (Y.H.H.); areum83@kbsi.re.kr (A.R.J.); 7 Department of Otolaryngology—Head and Neck Surgery, Yanbian University Hospital, Yanji 133000, China; jyd0091@126.com

**Keywords:** cochlea, round window membrane, RWM, noise-induced hearing loss, NIHL, sodium caprate, medium chain fatty acid, junctional modulation

## Abstract

Delivery of substances into the inner ear via local routes is increasingly being used in clinical treatment. Studies have focused on methods to increase permeability through the round window membrane (RWM) and enhance drug diffusion into the inner ear. However, the clinical applications of those methods have been unclear and few studies have investigated the efficacy of methods in an inner ear injury model. Here, we employed the medium chain fatty acid caprate, a biologically safe, clinically applicable substance, to modulate tight junctions of the RWM. Intratympanic treatment of sodium caprate (SC) induced transient, but wider, gaps in intercellular spaces of the RWM epithelial layer and enhanced the perilymph and cochlear concentrations/uptake of dexamethasone. Importantly, dexamethasone co–administered with SC led to significantly more rapid recovery from noise-induced hearing loss at 4 and 8 kHz, compared with the dexamethasone-only group. Taken together, our data indicate that junctional modulation of the RWM by SC enhances dexamethasone uptake into the inner ear, thereby hastening the recovery of hearing sensitivity after noise trauma.

## 1. Introduction 

The round window membrane (RWM) is located in the medial wall of the middle ear space and separates the inner ear from the middle ear [1,2]. The average thickness in humans is 70 µm and does not change with advancing age [2,3]. The membrane consists of three layers: an outer epithelial layer with a single layer of cells continuous with the mucous membrane lining the middle ear; a middle fibrous layer (i.e., connective tissue layer) containing lymph and blood vessels, fibroblasts, and collagen; and an inner epithelial cell layer consisting of several rows of thin cells, which are continuous with the mesothelial cells of the scala tympani [4]. The RWM serves as a barrier between the middle ear cavity and cochlea, conducts sound to the scala tympani, and participates in the secretion and absorption of substances. The permeability of the RWM is determined by multiple membrane factors (e.g., “compatibility determinants” for epithelial cell receptors) [1], including its size [5], configuration, concentration, liposolubility, electrical charge [6], and thickness [1,3,5]. Substances placed on the RWM traverse either diffusely through the cytoplasm as pinocytotic vesicles or through channels between cells in the epithelium [1,3]. In the middle fibrous (connective tissue) layer, cells pinocytize/phagocytize these substances, which then traverse toward the perilymph and/or penetrate blood or lymph vessels [1]. After the substances reach the perilymph, they are presumed to travel to the cerebrospinal fluid through the cochlear aqueduct, up to the scala tympani, or to the endolymph [3].

There is increasing clinical interest in the inner ear delivery of medication through local routes, instead of through systemic application, to bypass the blood–labyrinth barrier and achieve therapeutic drug levels in the inner ear while avoiding unwanted systemic side effects. Recent studies have focused on overcoming the anatomical barrier (biological membrane) to enhance drug diffusion into the inner ear. The permeability of the RWM is improved by benzyl alcohol, as well as solvents and detergents, such as dimethyl sulfoxide, *n*–methylpyrrolidone, and saponin [7]. However, the clinical application of these methods is unclear. For example, benzyl alcohol causes a burning sensation with intratympanic injection, and the other solvents and detergents listed above have not been investigated in terms of their toxicity to the inner and/or middle ear [7,8]. Thus, it is important to develop safe, clinically applicable, minimally invasive methods for intracochlear delivery to treat inner ear disorders. 

Here, we used a medium chain (C10) fatty acid, caprate, to modulate tight junctions in the RWM. Caprate is a well–recognized absorption enhancer that possesses antibacterial, antifungal, and anti–inflammatory properties. Caprate augments the permeation of macromolecules across tricellular tight junctions of intestinal cells and permeabilizes the blood–brain barrier [7,9,10]. Dexamethasone (dex), a synthetic glucocorticoid, is used for a wide range of medical conditions including inner ear diseases (e.g., Ménière’s disease, acute noise-induced hearing loss, and sudden idiopathic hearing loss). In the present study, dex was co-administered with sodium caprate (SC, capric acid sodium salt) through the intratympanic route. Then the perilymph and cochlear concentrations of dex were examined using a rat model.

## 2. Results 

### 2.1. SC Modulates Cellular Junctions of Round Window Membrane

**Figure 1:**
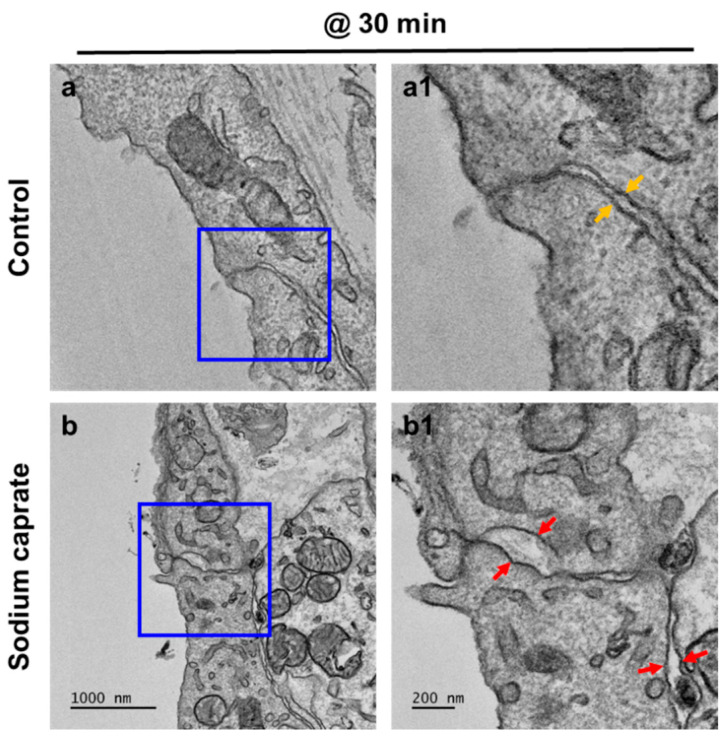
Ultrastructural analysis of the cochlear round window membrane in the control and intratympanic injection of the sodium caprate-treated group. Transmission electron microscopy (TEM) images of RWM were obtained at 30 min post-sodium caprate administration. (**a**) Control (untreated) animals showed normal appearing tight junctions which closely sealed the paracellular space between adjacent cells in the RWM outer epithelial layer (**a1**, yellow arrows). (**b**) Gaps in intercellular spaces (**b1**, red arrows) were observed at 30 min post-sodium caprate administration, while intracellular organelles appear normal and healthy. Sodium caprate (SC, 1.94 mg/ml, IT injection). *n* = 2. Scale bars indicate 1000 µm (**a**,**b**) and 200 µm (**a1**,**b1**).

**Figure 2:**
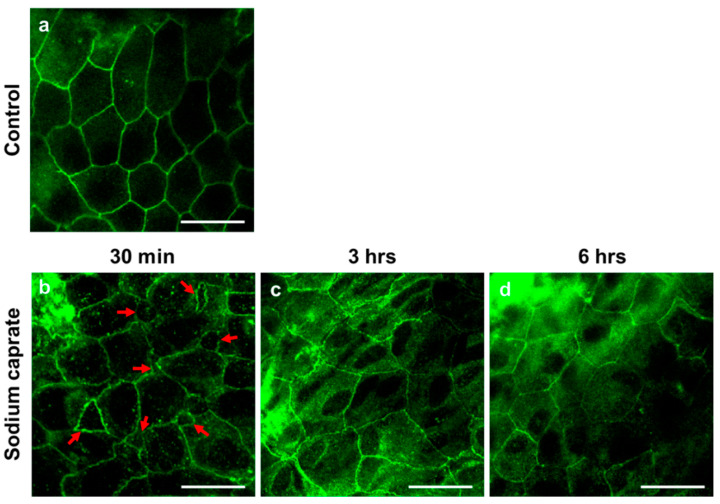
Representative confocal microscopy images of the cochlear round window membrane obtained from untreated (control) or sodium caprate-treated rats. Whole mounts of cochlear RWM were stained with *zonula occludens protein 1* (ZO–1, also known as tight junction protein–1, green fluorescence) antibody. (**a**) Control (untreated) rats showed no gaps between cells of RWM outer epithelial layer which create ”regular” pentagonal or hexagonal patterns on the surface. (**b**–**d**) Sodium caprate–treated animals presented irregular contours and gaps (red arrows) at 30 min (**b**), 3 h (**c**) and 6 h (**d**) post-sodium caprate administration. *n* = 2. Scale bar = 20 µm. All images were photographed from the middle ear side.

Male rats (7–8 weeks old) were randomly assigned into two groups (untreated control or SC [1.94 ug/ul, intratympanic injection]) and RWMs were obtained at 30 min post-treatment for ultrastructural analysis. Transmission electron microscopy images (Figure 1) revealed that control (untreated) animals had normal appearing tight junctions (Figure 1(a1), yellow arrows) in which the paracellular space was closely sealed with consistent distance between adjacent cells throughout the RWM outer epithelial layer. SC-treated rats had significantly wider gaps in intercellular spaces (Figure 1(b1), red arrows) than control rats. To further investigate whether SC modulates tight junctions on RWM epithelium, the zonula occludens (ZO)–1 protein was visualized from whole mounts of cochlear RWM. ZO–1 is a characteristic factor of tight junctions as it was the first tight junction protein to be cloned and used in previous studies [11,12,13]. At 30 and 60 min post-treatment (either control or SC), rats were euthanized and their cochleas were processed for histology. Confocal microscopy images of cochlear RWM were collected from untreated control-treated (Figure 2a) or SC-treated (Figure 2b,c) rats. Control (untreated) rats showed no gaps between cells of RWM outer epithelial layers which create ”regular” pentagonal or hexagonal patterns on the surface (Figure 2a). SC-administered animals presented “irregular” sizes and gaps (red arrows) at 30 min (Figure 2b) post-treatment. Importantly, the “irregular” gaps and morphologies of RWM become more regular and the gaps are rarely found at 3 h (Figure 2c) post-SC treatment and recover to control level at 6 h (Figure 2d). Taken together, these data indicate that intratympanic treatment of SC induces wider gaps in intercellular spaces of the RWM epithelial layer, however, which changes are transient, not permanent. 

### 2.2. Sodium Caprate Enhances Dexamethasone Uptake into Cochlea and Perilymph

**Figure 3:**
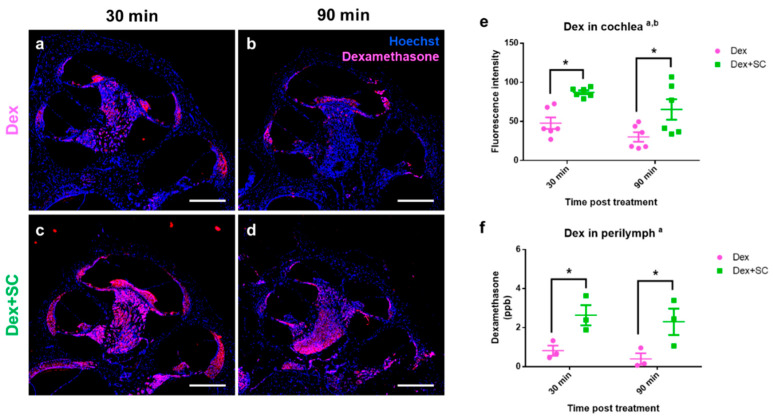
Dexamethasone uptake was enhanced by sodium caprate co-treatment in the cochlea as well as in the perilymph. (**a**–**d**) Cochlear tissues were collected from dex (**a**,**b**) and dex + SC (**c**,**d**) groups at 30 and 90 min post-treatment. Immunohistochemistry for dexamethasone (red) and nucleus (Hoechst, blue) staining revealed the presence of dexamethasone in the cochlear tissue after treatment. Sodium caprate co-treatment significantly increased dexamethasone uptake to the tissue at both time-points as compared to the dexamethasone-only group. Scale bar = 500 µm. (**e**) Intensity analysis confirmed that sodium caprate co-treatment significantly increased dexamethasone uptake to the cochlea at 30 and 60 min as compared to the dexamethasone-only group. Mean ± SEM. Two–way ANOVA; Tukey’s multiple comparisons test; a, main effect of treatment; b, main effect of time; *n* = 6; * *p* < 0.05. (**f**) Intracochlear perilymph was collected from the dex or dex + SC group at 30 and 90 min post-treatment, and the dexamethasone concentration was analyzed by LC–MS/MS. The sodium caprate-cotreated group showed significantly higher dexamethasone concentration in the perilymph at both time-points as compared to the dexamethasone-only group. Mean ± SEM. Two–way ANOVA; Fisher’s LSD; a, main effect of treatment; *n* = 3; * *p* < 0.05.

To examine whether SC-mediated junctional modulation affects dexamethasone uptake into the inner ear, 7- to 8-week-old male rats were randomly assigned into dex or dex + SC groups. At 30 and 90 min post-treatment, each cochlear tissue was collected from dex (Figure 3a,b) and dex + SC (Figure 3c,d) groups and processed for immunohistochemistry. Fluorescence microscopic images were collected from rat cochlea and stained against the dex (red) and nucleus (blue). Consistent with the junctional modulation by SC as shown in Figure 1 and Figure 2, dex uptake was significantly increased in dex + SC rats relative to the dex-only group at both time points (Figure 3c–e). Dex rats co–treated with SC (dex + SC) at both 30 and 90 min post-treatment showed greater dex-positive cells (Figure 3c,d) and increased fluorescence intensity (Figure 3e; a, main effect of treatment, F _(1, 20)_ = 20.65, *p* = 0.0002; b, main effect of time, F _(1, 20)_ = 5.814, *p* = 0.0256; Dex vs. Dex + SC at 30 min, *p* = 0.0138; at 90 min, *p* = 0.0313). 

Next, we measured the dex concentration from perilymphs using liquid chromatography/tandem mass spectrometry (LC–MS/MS) in dex or dex + SC treated rats collected at 30 and 90 min post-treatment. Dex concentration was significantly elevated in dex + SC-treated perilymphs relative to dex-treated perilymph samples (Figure 3f; a, main effect of treatment, F _(1, 8)_ = 15.77, *p* = 0.0041; Dex vs. Dex + SC at 30 min, *p* = 0.0255; at 90 min, *p* = 0.0206). Notably, both perilymph and cochlear levels of the dex quickly reached high peak values at 30 min in the dex + SC group (2.64 ± 0.52 ppb) compared to the dex-only group (0.82 ± 0.26 ppb). Taken together with the junctional modulation data (Figure 1), these data indicate that the SC co-treatment increases dexamethasone uptake to the inner ear at early time-points (~30 min), which lasts at least 90 min post-treatment.

### 2.3. Sodium Caprate Does Not Induce Hearing Impairment Nor Hair Cell Death

**Figure 4:**
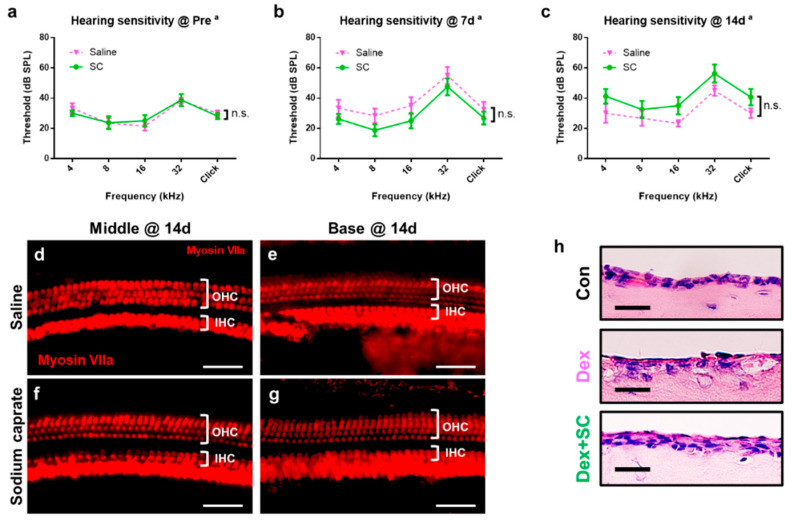
Sodium caprate displaying no sign of ototoxicity. (**a**–**c**) Auditory sensitivity was evaluated by the auditory brainstem response (ABR) in saline- or sodium caprate-treated rats at pre- (baseline, A) and post-treatment (7 and 14 d, **b**,**c**). Thresholds from ABR were recorded and graphed. No significant differences were found between groups treated with saline (pink line) and sodium caprate (green line) at all time points. Graphs represent mean ± SEM. Two-way repeated measures ANOVA; Sidak’s multiple comparisons test; a, main effect of frequency; n.s., not significant; *n* = 6 (saline); *n* = 8 (sodium caprate). (**d**–**g**) Sodium caprate does not alter cochlear hair cells. Microdissected cochlear tissues were immunofluorescently stained for myosin-VIIa and imaged using epifluorescence. Representative images were shown from the cochlear middle and basal turns to show the hair cell densities for the three rows of OHC and single row of IHC for the saline-treated control (**d**,**e**) and that treated with sodium caprate (**f**,**g**). No significant differences were observed between the saline- and sodium caprate-treated groups at 14 d from both the middle and basal turns. Scale bar = 100 µm. (**h**) Dex + SC-treated mucosa presents healthier lining and morphology as compared to the dex-only group, indicating SC induces no sign of damage at 14 d post-treatment. Scale bar = 15 µm.

To investigate whether the SC per se has any potential harm on the inner ear, hearing sensitivity and hair cell survival/death were measured in saline- or SC-treated rats at pre- and post-treatments. Auditory brainstem response (ABR) threshold analysis demonstrates that there was no significant differences between saline- and SC-treated rats, exhibiting an insignificant main effect of treatment (*p* > 0.05) and a significant main effect of frequency (*p* < 0.0001) pre-treatment (Figure 4a), and at 7 d (Figure 4b), and 14 d (Figure 4c) post-treatment. Similar to the ABR threshold, inner (IHCs) and outer hair cells (OHCs) from both saline- (Figure 4d,e) and SC-treated rats (Figure 4f,g) showed no sign of loss or death at 14 d post-SC treatment. 

To investigate whether intratympanically administered SC have any adverse impact in the middle ear, middle ear mucosa was collected and examined. As the dex is widely used and approved as an intratympanic treatment, dex + SC-treated mucosa was compared to the dex-treated middle ear mucosa. Figure 4h confirms that dex + SC induced healthier mucosa lining and morphology as compared to dex alone, although both dex and dex + SC presented with more damage than the untreated control middle ear mucosa. 

These data suggest that junctional modulation by SC in rats does not significantly impact auditory function measured by the ABR threshold and hair cell survival shown by immunostaining at 14 d post-SC treatment. 

### 2.4. Sodium Caprate Co-Treatment Hastens Recovery from Noise-Induced Hearing Loss

**Figure 5:**
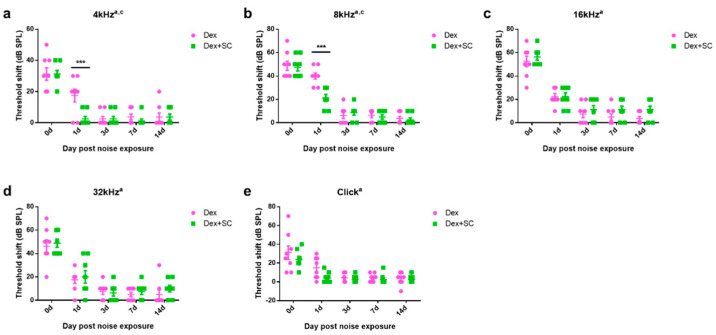
Sodium caprate co-treatment hastens recovery from noise trauma. (**a**,**b**) At 4 and 8 kHz, SC co-treatment significantly hastened recovery at 1 d post-noise trauma as compared to the dex-only group. (**c**–**e**) No significant differences were found from other frequencies and click. Graphs represent mean ± SEM. Two-way repeated measures ANOVA; Sidak’s multiple comparisons test; a, main effect of treatment; c, main effect of interaction; *** *p* < 0.001.

To test whether the enhanced dex uptake by junctional modulation actually affects the functional recovery from noise trauma, all rats were randomly assigned into two groups of dex only or the dex + SC group and exposed to free-field broadband noise (1 kHz–8 kHz) for 5 min at an intensity of 115 dB SPL. Hearing sensitivity was measured by ABR, and the threshold shift is graphed in Figure 5. At 4 and 8 kHz, SC co-treatment significantly hastened recovery at 1 d post-noise trauma as compared to the dex-only group (Two-way repeated measures ANOVA; Sidak’s multiple comparisons test; a, main effect of treatment; c, main effect of interaction; ***, *p* < 0.001). No significant differences were found from other frequencies and click. 

**Figure 6:**
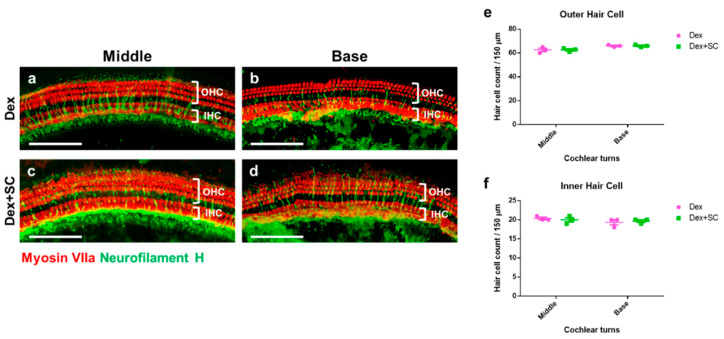
Cochlear hair cell survival between dex and dex + SC groups. The whole mount of microdissected cochlear tissues were immunofluorescently stained for myosin-VIIa (red) and neurofilament H (green, Neurofilament) and photographed using epifluorescence. Representative images were shown from the cochlear middle and basal turns to show the dex-treated (**a**,**b**) and dex + SC- treated (**c**,**d**) hair cell survival. No differences were found between the inner (**e**) and outer (**f**) hair cell counts between dex- and dex + SC-treated groups at 14 d from both middle and basal turns. Scale bar = 100 µm. Mean ± SEM; Two-way ANOVA; *n* = 4.

Whole-mount staining of the inner (IHCs) and outer hair cells (OHCs) from both dex- and dex + SC-treated rats did not show cellular loss or death at 14 d post-noise trauma (Figure 6a–d), confirmed by quantification of OHCs (Figure 6e) and IHCs (Figure 6f). These data suggest that junctional modulation by SC in rats at least partially improves auditory sensitivity and hastens recovery from noise trauma. 

## 3. Discussion

**Figure 7:**
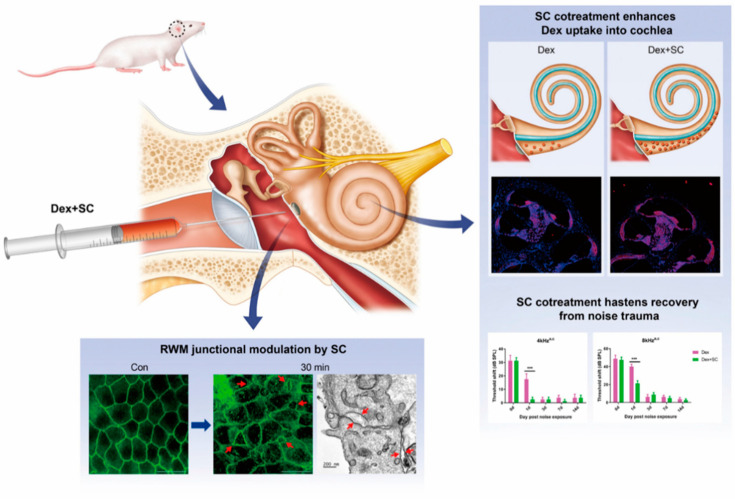
RWM junctional modulation improves dexamethasone uptake into the inner ear and shows faster recovery after noise-induced hearing loss. Diagram of the summary by which SC cotreatment increases dexamethasone concentration into the perilymph and cochlea. Junctional modulation by SC cotreatment in rats, therefore, improves hearing and hastens recovery from noise trauma. *** *p* < 0.001. a, main effect of treatment; c, main effect of interaction.

The present study showed (Figure 7) that by local intratympanic application of dex with SC, the perilymph concentration quickly peaked within 30 min of treatment. Consistent with the perilymph concentration, dex uptake into the cochlea was also significantly enhanced by SC as compared to the dex-only group. Ultrastructural images revealed that SC modulated cellular junctions, which was shown to be transient, also evidenced by our previous work [14], without affecting hearing sensitivity and hair cell survival. 

According to the American Academy of Otolaryngology–Head and Neck Surgery Foundation’s Clinical Practice Guideline Development Manual [15] and an updated review [16], the guideline update group offered optional use of corticosteroids as initial therapy to patients with sudden sensorineural hearing loss within 2 weeks of symptom onset (key action statement 8). Dexamethasone has been widely applied to treat sudden hearing loss and inner ear injuries and the most widely used route is still systemic administration in humans [17]. The risk of inducing undesirable adverse effects is associated with systemic dexamethasone administration, including hypothalamic–pituitary–adrenal axis suppression [18], the Cushing phenomenon [19], and avascular necrosis of the femoral head [20]. These potentially detrimental side-effects limit their clinical use for treatment of inner ear diseases by systemic administration. Thus, it is important to find a safe local delivery method for drug administration. The main advantage is that the local intratympanic application can obtain higher levels of inner ear concentration and reduced systemic toxicity because the drug bypasses the blood–labyrinth barrier and enters the inner ear directly through RWM. 

Tight junctions form the intercellular barrier. In the epithelial layer of the RWM, tight junctions are composed of the transmembrane and cytoskeletal and cytoplasmic plaque proteins, such as the claudins, occludin, junction adhesion molecules, and ZO–1 [21]. ZO–1 is known to modulate the structure of tight junctions by interacting with transmembrane factors (e.g., claudins, occluding and junction adhesion molecules) [22]. We have previously reported reduced ZO–1 density and increased paracellular space by SC in the cochlear auditory epithelium of guinea pigs, of which changes were recovered similarly to the control ear by 4 h post-SC treatment [14]. While we, at least partially, verified the safety of SC to the inner ear by both functional (ABR threshold) and histopathological (hair cell survival) methods in the present study, we have not fully tested the pharmacokinetics of SC in the inner ear. It can be speculated that SC (and dex) may be removed by passive diffusion to endolymph/CSF through the cochlear aqueduct and by active elimination, such as blood flow and lymphatic flow [3,23]. Although the safety (e.g., perilymph fistulas, foreign body reactions) of SC should be further investigated for potential clinical use of SC in the inner ear, our data provide the first evidence that intratympanically injected SC have no adverse effect of hair cell loss/survival and hearing sensitivity at 14 days post-treatment, while SC significantly enhances dex uptake into the inner ear in a rodent model. 

Although recent studies have focused on ways to augment substance delivery into the inner ear, the previously reported methods [7,8] still need further consideration for the clinical approach. As reported by others and mentioned in our current study, when dex was administered via the intratympanic route, its concentration in the perilymph was not high enough to achieve therapeutic drug concentration in the inner ear, even though the levels were higher in local administration than in systemic (intraperitoneal) application [17,24]. This may explain why a significant number of patients with sudden sensorineural hearing loss hardly recovers from the illness, even after immediate intratympanic dex (steroid) administration [25]. Considering the fact that once a substance in the middle ear passes through the RWM there are no more physiological barriers to reach the target cells in the cochlea, our data may suggest an effective way of developing safe, clinically applicable methods for intracochlear drug delivery to treat inner ear disorders.

The effect of increased bioavailability of dex in the inner ear specifically is unknown in an injury model. Previous fascinating works proved the possibilities to modulate RWM permeability [26,27,28]; however, there are currently no data indicating the effectiveness of junctional modulation in a model of inner ear injury. In the current study, a transient threshold shift (TTS) was utilized to mimic the noise trauma model in humans, where SC co-treatment showed faster recovery from noise trauma relative to the dex-only group. The protective effect of SC was prominent at the early acute phase 1 d post-noise trauma, at 4 and 8 kHz. This may be because the SC’s impacts on RWM junctional modulation were most potent (but transient) at the early phase after treatment. Our data indicate that SC treatments are effective at increasing RWM permeability without affecting the hearing sensitivity nor cochlear hair cell survival, at least in our model, and to ameliorate the noise-induced hearing loss. 

Over the past decade, drug delivery to the inner ear emerged; thus, the biological barrier RWM has become one of the key targets for the modulatation of substance permeability. Previous trials include osmotic pumps [29], constant infusion intracochlear delivery systems [30], and the cochlear prosthesis-mediated drug delivery system [31,32,33,34]. However, how they might be used in clinical applications is still unclear [3]. Compared to the above-mentioned methods, RWM junctional modulation by SC seems less invasive and clinically more applicable by intratympanic injection. Moreover, SC treatment can be easily combined with novel or already–proven biomaterials and biotechnology. For instance, SC treatment followed by dexamethasone administration packaged in hydrogels and/or nanoparticles can not only enhance drug delivery into the inner ear, but also sustain/control the release of drugs into the cochlea. Besides the RWM junctional modulation, pharmacokinetics of the drugs should be further investigated to establish the toxicity and safety of new inner ear drug application. As an already clinically used class of drugs, SC may offer a unique opportunity to safely enhance drug delivery in patients undergoing inner ear diseases, especially sudden hearing loss. 

## 4. Materials and Methods

### 4.1. Animals

Forty-six 7-week-old male Sprague–Dawley rats were purchased from Samtaco (Osan, Korea) and maintained in a temperature- (22 °C) and humidity- (45–55%) controlled environment with a 12/12 h dark–light cycle (from 7:00 a.m. to 7:00 p.m.). Rats were fed pelleted food (Envigo, #2018 Teklad Global 18% Protein Rodent Diet) and water ad libitum. All animal procedures were approved by the IACUC of Chungnam National University (protocol number CNU01116). 

### 4.2. Intratympanic Injection

Rats were anesthetized with alfaxan (10 mg/kg, intramuscular injection, Careside, Sungnam, Korea) and xylazine (10 mg/kg, intramuscular injection, Bayer Animal Health, Monheim, Germany) and maintained at 37 °C on a heating pad. A retroauricular incision was made bilaterally; the temporal bone was exposed and opened to visualize the round window membrane under a surgical microscope (Carl Zeiss OPMI f 170 Surgical Tilting Head connected to a Hitachi KP–D50 Color Digital Microscope Camera). Dexamethasone sodium phosphate (5 mg/ml, Huons, Sungnam, Korea) or sodium caprate (1.94 mg/ml, Sigma–Aldrich, St. Louis, MO, USA) was administered into tympanic cavity using an insulin syringe (85 µL/injection) connected to the tip of a 31-gauge needle (BD, Seoul, Korea). After injection, the needle was carefully removed, and dental cement (Durelon™ Carboxylate Luting Cement, 3 M, Seoul, Korea) was applied to the area.

### 4.3. Transmission Electronic Microscopy (TEM)

Rat cochlea was prepared for TEM as described previously [35]. Briefly, the decalcificated rat cochlea were prefixed immediately in 2.5% glutaraldehyde 2% paraformaldehyde in 0.15 M sodium cacodylate buffer (pH 7.4) for 2 h at 4 °C. After washing with sodium cacodylate buffer, rat tissue samples were postfixed in 2% osmium tetroxide and 1.5% ferrocyanide in 0.15 M cacodylate buffer (pH 7.4) for 1 h. Samples were incubated with 1% TCH for 30 min and treated with 2% OsO_4_ for 30 min, then En bloc stained with 1% uranyl acetate overnight at 4 °C and lead citrate for 30 min at 60 °C. The tissues were then embedded in an Epon 812 mixture after dehydration in an ethanol and propylene oxide series. Polymerization was conducted with pure resin at 70 °C for 24 h. Sections (200 nm) were obtained with an ultramicrotome (Ultra Cut–UCT, Leica, Vienna, Austria) and then collected on 100 mesh copper grids. The sections were visualized using conventional TEM (JEM–1400Plus) at 120 kV and Bio–HVEM (JEM–1000BEF, JEOL, Tokyo, Japan) at 1000 kV. The sections were visualized using a Bio–HVEM system (JEM–1400Plus at 120 kV and JEM–1000BEF at 1000 kV, JEOL, Japan).

### 4.4. Immunostaining

Primary antibodies and dilutions were used as follows: Mouse anti-ZO-1 (Invitrogen, 1:100), Rabbit anti-dexamethasone (Abcam, 1:200), Hoechst 33342 (Invitrogen, 1:2000), Rabbit anti-Myosin VIIa (Proteus BioSciences, 1:200), and Chicken anti-Neurofilament H (Sigma–Aldrich, 1:200). AlexaFluor 488 anti-mouse secondary antibody (Molecular Probes, 1:200), AlexaFluor 594 anti-rabbit secondary antibody (Invitrogen, 1:200), AlexaFluor 488 anti-chicken secondary antibody raised in goat (Invitrogen, 1:200) were used. For immunostaining, tissues were dissected, washed three times with phosphate buffered saline (PBS), fixed with 4% paraformaldehyde (PFA) in PBS overnight at 4 °C and washed three times in PBS, and then blocked with 10% normal chicken serum in 0.3% Triton X-100 for 1 h. 

For whole-mount immunostaining, dissected cochleae or round window membrane (RWM) were permeabilized and blocked at room temperature for 1 h in PBS containing 0.3% Triton and 1.5% normal goat serum. The primary antibody was incubated in the same blocking solution overnight at 4 °C, followed by three washes in PBS at room temperature. The secondary antibody was incubated for 2 h and washed in PBS at room temperature. Prior to imaging, tissues were mounted on glass slides using CrystalMount (Biomeda, Foster City, CA, USA) and imaged using a confocal microscope (Leica Microsystems, Wetzlar, Germany).

For section immunostaining, 4% PFA-fixed, and 10% EDTA-decalcified, paraffin-embedded tissues were prepared and stored at −80 °C prior to sectioning at 4 µm thickness. Blocking and antibody hybridization steps were carried out in PBS containing 1.5% normal chicken serum and 0.3% Triton X-100. Section immunostaining was observed by epifluorescence microscopy (BX53F2, Olympus, Tokyo, Japan).

### 4.5. Detailed Antibody Information Are as Follows

ZO–1, Thermo Fisher Scientific, catalog number (33–9100), lot number (TL277395), clone number (zo1–1A12);

MyoVIIa, Proteus, catalog number (25–6790), lot number (110119), clone number (not available);

Neurofilament H, Sigma–Aldrich, catalog number (AB5539), lot number (3328929), clone number (not available);

Dexamethasone, Abcam, catalog number (ab35000), lot number (GR246267–19), clone number (not available);

Hoestch 33342, Thermo Fisher Scientific, catalog number (H3570), lot number (1387197), clone number (not available).

### 4.6. Cochlear Perilymph Collection and Dexamethasone Concentration Measurement

Under anesthesia, a retroauricular incision was made bilaterally and the tympanic bulla was washed with saline. A small apical cochleostomy was performed under a surgical microscope (Carl Zeiss OPMI f 170 Surgical Tilting Head connected to a Hitachi KP–D50 Color Digital Microscope Camera). Perilymph was collected from the cochlear apex in capillary tubes (Sigma–Aldrich, St. Louis, MO, USA). Cochlear perilymph samples and standards were diluted and analyzed using an LC–MS/MS method equipped with an Agilent 1290 Infinity II LC system (Agilent Technologies, Santa Clara, CA, USA) and a QTRAP^®^ 6500 LC–MS/MS System (SCIEX, Framingham, MA, USA). 

For liquid chromatographic separation, an Agilent Eclipse XDB–C18 (50 mm × 2.1 mm, 3.5 μm, Agilent Technologies, Santa Clara, CA, USA) was applied with a column temperature of 40 °C. The mobile phase consisted of solvent A (0.1% formic acid in deionized water) and solvent B (methanol) at a flow rate of 0.4 ml/min. The autosampler was maintained at 5 °C with an injection volume of 10 μl for all samples. 

For mass spectrometry separation, a QTRAP^®^ 6500 LC–MS/MS System (SCIEX, Framingham, MA, USA) equipped with electrospray ionization (Turbo VTM ion source) was used. Data acquisition was performed in a positive multiple reaction monitoring (MRM) mode with a duration of 6 min and a total cycle of 774. The ion source potential and collision energy were optimized for each MRM transition. The decluttering potential (DP) was 36 V, the entrance potential (EP) was 10 V, the collision energy (CE) was 10 V, and the cell exit potential (CXP) was 12 V. Nitrogen was used as curtain gas (35 psi), ion source gas 1 (nebulizer gas, 50 psi), and ion source gas 2 (turbo gas, 50 psi). The ion source temperature was 500 °C and ion source voltage was 5500 V.

### 4.7. Noise Exposure

In the noise exposure group, animals were exposed to free-field broadband noise (1 kHz–8 kHz) for 5 min at an intensity of 115 dB SPL. The noise signals were routed through a computer and an amplifier (INTER–M R300 Plus power amplifier; Canford Audio PLC, Washington, UK) to a loudspeaker (ElectroVoice DH1A–WP; Sonic Electronix Inc., Sylmar, CA, USA). The noise level was measured using a sound level meter (B&K type 2250; Brüel and Kjaer, Naerum, Denmark), sound calibrator (B&K type 4231; Brüel and Kjaer), and condenser microphone (B&K type 4189; Brüel and Kjaer).

### 4.8. Auditory Brainstem Response (ABR)

ABR thresholds at frequencies between 4 and 32 kHz and click sounds were obtained separately from both ears. The TDT System–3 (Tucker Davis Technologies, Gainesville, FL, USA) hardware and software were used to obtain the ABRs. The stimuli were computer-generated tone pips. The animals were anesthetized with intramuscular injection of zolazepam HCl 40 mg/kg (Zoletil, Virbac Animal Health, Carros, France) and xylazine 10 mg/kg (Rompun, Bayer Animal Health, Monheim, Germany). Subcutaneous needle electrodes were placed around the skull vertex and both infraauricular areas. Tone bursts, with a duration of 4 ms and rise–fall time of 1 ms at frequencies of 4, 8, 16, and 32 kHz were used, in addition to clicks. The sound intensity was varied in 5 dB increments for the tone burst sounds and clicks. The contralateral ear was not masked because the stimuli were transmitted through a sealed earphone. The waveforms were analyzed using a custom program (BioSig RP, ver. 4.4.1; Tucker Davis Technologies) with the researcher blinded to the treatment group. The threshold was defined as the lowest stimulus intensity to evoke a wave III response > 0.2 μV.

### 4.9. Statistics

Data graphing and all statistical analyses were performed using GraphPad Prism 6 (GraphPad Software, San Diego, CA, USA). All measurements were taken from distinct samples and sample sizes were determined without any expectation of the effect size. All experiments were repeated multiple times, and the numbers of individual measurements are included in the main body of the text and/or in the figure legends. Two-way ANOVA was used for dexamethasone concentration measurements and hair cell counts. For ABR, a two-way repeated measures ANOVA coded for treatment and day/time was used. All data comparisons were performed after assessment of normality and variance. Group differences were considered significant at *p* < 0.05 in each case. All data were expressed as the mean  ±  SEM. All images and data were first coded, and all analyses were performed by an investigator blinded to the codes.

## Data Availability

The datasets generated during and/or analysed during the current study are available from the corresponding author on reasonable request.
